# ﻿Taxonomic notes on *Sorbusmegalocarpa* (Rosaceae) and related taxa

**DOI:** 10.3897/phytokeys.201.84027

**Published:** 2022-06-30

**Authors:** Xin Chen, JianHui Ma, LiYang Geng

**Affiliations:** 1 Co-Innovation Center for Sustainable Forestry in Southern China, College of Biology and the Environment, Nanjing Forestry University, Nanjing 210037, China Nanjing Forestry University Nanjing China

**Keywords:** resurrection, *Sorbus*, synonymization, taxonomy

## Abstract

Four *Sorbus* taxa endemic to China, *S.arguta*, *S.guanxianensis*, S.megalocarpavar.megalocarpa and S.megalocarpavar.cuneata, are morphologically similar to one another in having large brown fruits with persistent calyx and dense lenticels. In literature, either all of the four taxa were accepted, or two of them, *S.arguta* and S.megalocarpavar.cuneata, were treated as synonyms of S.megalocarpavar.megalocarpa, or *S.guanxianensis* alone was dubious. In this study, based on morphological comparison, *S.arguta* is reinstated for its relatively small inflorescence, small fruit and timing of flowering after leaves are unfolded. S.megalocarpavar.cuneata is confirmed as a synonym and *S.guanxianensis* is proposed as a new heterotypic synonym of *S.megalocarpa*.

## ﻿Introduction

*Sorbus* L. (1753: 477; Rosaceae) in the broad sense (*sensu lato*, *s.l.*) comprises about 100 to more than 250 species mainly distributed in northern temperate regions with the center of diversity in China (Phipps et al. 1990; Lu and Spongberg 2003). Although previous molecular studies indicated that *Sorbuss.l.* is highly polyphyletic with species falling into six genera: *Aria* (Pers.) Host, *Chamaemespilus* Medik. (1879: 138), *Cormus* Spach, *Micromeles* Decne. (1874: 168), *Sorbus* and *Torminalis* Medik. (1874: 134; Campbell et al. 2007; Li et al. 2012; Lo and Donoghue 2012; Sun et al. 2018; Ulaszewski et al. 2021), the taxonomic diversity of *Sorbus* in China is being included within a single genus (Yü and Lu 1974; Lu and Spongberg 2003). The number of species native to China recognized varies tremendously according to different taxonomists (Yü and Lu 1974; Phipps et al. 1990; Lu and Spongberg 2003; Aldasoro et al. 2004; McAllister 2005). For example, in the latest revision of Sorbussubg.Aria Persoon and *Torminaria* (DC.) Reichenbach, Aldasoro et al. (2004) accepted only 21 species out of the total 31 species and 6 varieties recognized by Lu and Spongberg (2003). The striking inconsistencies in taxonomic treatments have given rise to confusion in species identification and utilization. The controversial delimitation of *S.megalocarpa*Rehder (1915: 266) and its allies, S.megalocarpavar.cuneataRehder (1915: 267), *S.arguta* T. T. Yü (Yü and Kuan 1963: 223) and *S.guanxianensis*Ku (1990: 22), is an example here. The four taxa were all accepted by Yü and Lu (1974), Lu and Spongberg (2003). *S.arguta* and S.megalocarpavar.cuneata were treated as synonyms of *S.megalocarpa* and *S.guanxianensis* was regarded as a doubtful species by Aldasoro et al. (2004). The purpose of this paper is to clarify the taxonomic confusion and to enhance stability of these names based on protologues, related literature studies, original materials examinations and field investigations.

## ﻿Materials and methods

Type collections and voucher specimens of *Sorbusarguta*, *S.guanxianensis*, S.megalocarpavar.megalocarpa and S.megalocarpavar.cuneata were examined from the following herbaria: A, CDBI, E, GH, IBSC, KUN, NF, PE, WCSBG and US (acronyms follow Thiers continuously updated); virtual images were examined mainly through the website PPBC (http://ppbc.iplant.cn/). Morphological comparison presented here is based on analysis of specimens, as well as fresh materials collected by ourselves.

## ﻿Taxonomic treatments

### ﻿1. Sorbus megalocarpa

Taxon classificationPlantaeRosalesRosaceae

Rehder, Pl. Wilson. 2(2): 266. 1915.

00CBB6EF-CDAD-51A6-9179-3CA50361BADA

≡ Ariamegalocarpa (Rehder) H. Ohashi et Iketani, J. Jap. Bot. 68(6): 359. 1993.
≡ Micromelesmegalocarpa (Rehder) Mezhenskyj, NULESU Coll. Fruit Ornament. Pl.: 34. 2018.
≡ Wilsonariamegalocarpa (Rehder) Rushforth, Phytologia 100(4): 241. 2018.
= Sorbusmegalocarpavar.cuneata Rehder, Pl. Wilson. 2(2): 267. 1915. Type: CHINA. Sichuan: Western Szechuan, Mupin, 2400–2700 m, 10 October, 1910–11, *E.H. Wilson 4215* (lectotype, designated by Aldasoro et al. 2004, pg. 43: K[K000758157]; isolectotype: A[A00112653])
= Ariamegalocarpavar.cuneata (Rehder) H. Ohashi et Iketani, J. Jap. Bot. 68(6): 359. 1993.
= Sorbusguanxianensis T.C. Ku, Bull. Bot. Res., Harbin 10(3): 22, f. 2. 1990. syn. nov. Type: China. Sichuan: Guanxian (Dujiangyan), 2000 m, 25 August 1987, T.Z. *Fu et al. 872102* (holotype: PE[PE00020830]), syn. nov.
= Micromelesguanxianensis (T.C. Ku) Mezhenskyj, NULESU Coll. Fruit Ornament. Pl.: 34. 2018. syn. nov.
= Wilsonariaguanxianensis (T.C. Ku) Rushforth, Phytologia 100(4): 241. 2018. syn. nov.

#### Type.

China. Sichuan: Western Szechuan, Hung-yah Hsien (Hongyaxian), 1200 m, 12 September 1908, *E.H. Wilson 956* (lectotype, designated by Gabrielian 1978, pg. 220: K[K000758158]; isolectotypes: A[A00112650], E[E00147452], GH[GH00112651], US[US00097467]).

#### Notes.

***Sorbusmegalocarpa***: *Sorbusmegalocarpa* was first published by Rehder (1915: 266). It was transferred to genera *Aria*, *Micromeles* and *Wilsonaria* by Ohashi and Iketani (1993: 359), Mezhenskyj (Mezhenska et al. 2018: 34) and Rushforth (2018: 241) respectively.

**Figure 1.**
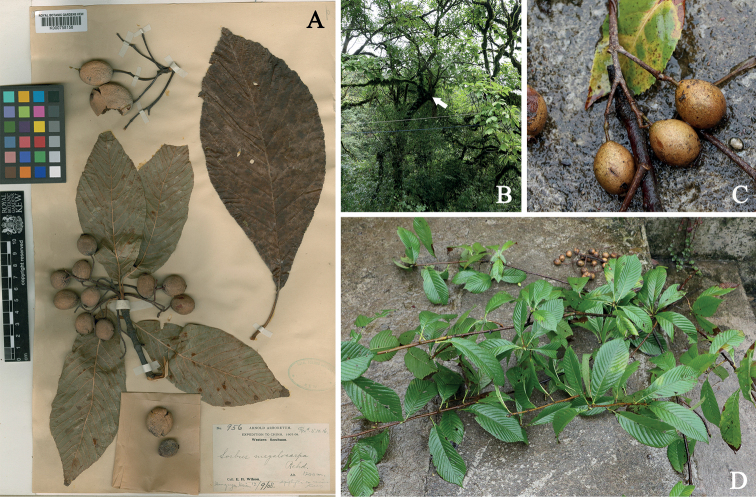
*Sorbusmegalocarpa* Rehder **A** lectotype (K000758158) **B** an epiphytic plant in the wild (Ya’an, Sichuan, China) **C** fruit (showing the color of fruit and lenticels on it) **D** leaves (showing the variation of leaf shape and petiole length in one plant).

Three gatherings collected by Wilson under number “956” were cited in the protologue. The first one was collected at alt. 2200–2600 m., Mupin, in October 1910; the second one was collected at alt. 1200 m., Hung-ya Hsien, on September 12, 1908; and the third one was collected at alt. 2000 m., Mon-kong Ting, on June 19, 1908. Since the author did not indicate holotype for the name, the three gatherings are syntypes according to the Article 9.6 of the *International Code of Nomenclature for algae*, *fungi and plants* (*Shenzhen Code*) (Turland et al. 2018). Gabrielian (1978) designated the specimen at K (K000758158; as shown in fig. 1A, plate 62 of Gabrielian 1978), collected from Hung-Ya Hsien, as the lectotype. Four duplicates (A00112650, E00147452, GH00112651, US00097467) out of the eight specimens of “*E. H. Wilson 956*” traced at A, E, GH, K and US, are the isolectotypes here.

**Sorbusmegalocarpavar.cuneata**: Rehder (1915: 267) differentiated Sorbusmegalocarpavar.cuneata from S.megalocarpavar.megalocarpa by its “smaller softer fruit” (“fructibus minoribus ovovides circiter 1.5 cm. longis et 1 cm. diam.” and “the more cuneate short-stalked leaves” (“petiolum vix 1 cm”). This variety was recognized by Yü and Lu (1974) and Lu and Spongberg (2003), was transferred to *Aria* by Ohashi and Iketani (1993: 359) and was treated as a synonym of *S.megalocarpa* by Aldasoro et al. (2004). The great variability of leaves and fruits of *S.megalocarpa* was well documented by Aldasoro et al. (2004) and confirmed in our field investigations (Fig. 1B–D). *Sorbusmegalocarpa* has elliptic, elliptic-obovate, obovate-oblong leaves with crenate-serrate margins and petiole of 0.7–2 cm long, and large ovoid, ovoid-globose, or sub-globose fruits (1–2.7 cm long, 0.7–2.2 cm in diameter) covered with dense lenticels. The length of petiole and size of fruits of S.megalocarpavar.cuneata are within the variation range of *S.megalocarpa*. Therefore, we agree with Aldasoro et al. (2004) in reducing S.megalocarpavar.cuneata to a synonym of *S.megalocarpa*.

**Figure 2.**
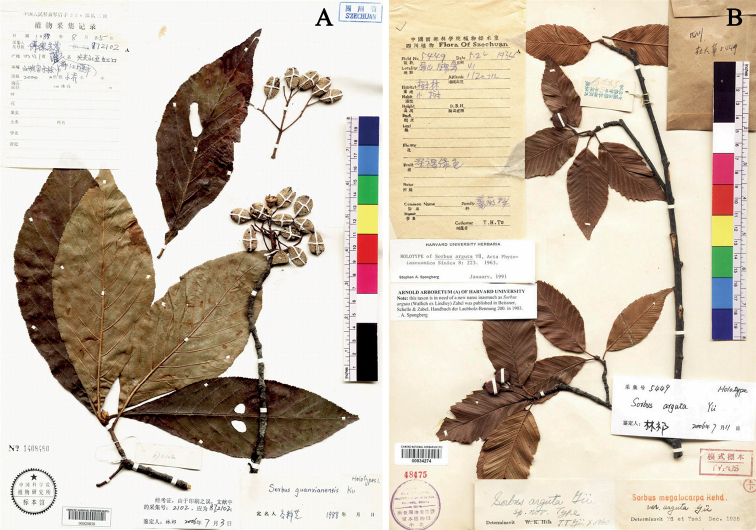
**A** holotype of *Sorbusguanxianensis* Ku (PE00020830) **B** holotype of *Sorbusarguta* T. T. Yu (PE00934274).

***Sorbusguanxianensis***: Ku (1990: 22) published *Sorbusguanxianensis* based on two gatherings, “*T. Z. Fu et al 2102*” (Fig. 2A) and “*Z. L. Zhao 0970*”. In the protologue, Ku (1990) included the diagnostic words “calycis lobi mox decidui”, compared it with *S.alnifolia* (Siebold and Zuccarini) K. Koch in Sorbussect.Micromeles and differed it by its larger fruits (about 1.5 cm long), though she assigned it to Sorbussect.Aria (Ku 1990). This contradictory taxonomic description led later authors to treat *S.guanxianensis* in different circumscriptions. Phipps et al. (1990) and Lu and Spongberg (2003) accepted it. Mezhenska et al. (2018: 34) and Rushforth (2018: 241) also recognized it and transferred it to *Micromeles* and *Wilsonaria* respectively. Aldasoro et al. (2004) considered it “a doubtful species” and stated that pomes of *S.guanxianensis* “without lenticels” and “may by a synonym of *S.zahlbruckneri*”.

No specimens of *Sorbusguanxianensis* other than the two gatherings cited in the protologue are available in herbarium. Our examination of the type specimens indicated that characters such as persistent calyx and dense lenticels on pomes were in serious conflict with the description in the protologue and were neglected by Aldasoro et al. (2004). *Sorbusguanxianensis* could easily be distinguished from *S.zahlbruckneri* C. K. Schneider by the leaves which have margins “singly dentate (not double-dentate)” as stated by Aldasoro et al. (2004) themselves. The unusual characters possessed by the type specimens were noted by Rushforth (2018) who stated that *S.guanxianensis* “seems to match Rehder’s S.megalocarpavar.cuneata” and transferred it to *Wilsonaria* together with *S.megalocarpa*. Morphological similarities (Styles 3 or 4, leaves glabrous or sparsely hair when young, not tomentose, fruit brown, 12–20 mm in diameter, covered in massed contiguous lenticels) stated by Rushforth (2018), and a detailed critical read of the protologues and evaluation of the specimens confirmed that *S.guanxianensis* is conspecific with *S.megalocarpa*. Accordingly, we proposed to reduce *S.guanxianensis* as a heterotypic synonymy of *S.megalocarpa* here.

#### Representative specimens examined.

China. Sichuan: Baoxin county, 17 July 1925, *K.L. Chu 3149* (IBSC); Dayi county, Xiling town, Chadiping, Xiling Snow Mountain, 30°38'24.84"N, 103°09'52.33"E, 1471 m, 1 June 2015, *J.D. Ya and X. J. Hu 15CS11089* (KUN); Dujiangyan, Hongkou town, Dashuigou conservation station, 1250 m, *D.H. Zhu*, *C. Zhang*, *X.J. Li 4872* (WCSBG); Hongya county, forest farm, July 1992, *Z.W. Wang A00088* (CDBI); Hongya county, Lewu town, Shuanghekou, 2100–2230 m, 2 August 1959, *Z.T. Guan 9120* (PE); Hongya County, Lewu town, Shuanghekou, 2100–2230 m, 3 August 1959, *Z.T. Guan 6814* (PE); Leibo county, Mahu town, 1300 m, 25 May 1959, *238 collection team 0338* (PE); Leibo county, Shahezhou forest farm, 2400 m, 12 August 1972, *238 collection team 0697* (PE); Ya’an city, Yingjing county, Longchigou National Forest Park, Daxiangling, 29°36'21.23"N, 102°50'28.48"E, 1364 m, 19 September 2020, *X. Chen*, *X.Y. Wang*, *C.H. Wang 1891* (NF); Ya’an city, Yingjing county, Longcanggou National Forest Park, Diecuixi, 29°36'55.01"N, 102°53'42.57"E, 1509 m, 19 September 2020, *X. Chen*, *X.Y. Wang*, *C.H. Wang 1906* (NF); Ya’an city, Yingjing county, Longcanggou National Forest Park, Diecuixi, 29°36'57.42"N, 102°53'38.74"E, 1512 m, 19 September 2020, *X. Chen*, *X.Y. Wang*, *C.H. Wang 1907* (NF); Ya’an city, Yingjing county, Longcanggou town, Fazhan village, 29°37'04.96"N, 102°53'25.17"E, 1466 m, 20 September 2020, *X. Chen*, *X.Y. Wang*, *C.H. Wang 1908* (NF); Ya’an city, Yingjing county, Longcanggou town, Fazhan village, 29°38'15.34"N, 102°53'00.64"E, 1359 m, 20 September 2020, *X. Chen*, *X.Y. Wang*, *C.H. Wang 1914* (NF); Ya’an city, Yingjing county, Longcanggou town, Fazhan village, 29°37'48.37"N, 102°53'13.75"E, 1358 m, 20 September 2020, *X. Chen*, *X.Y. Wang*, *C.H. Wang 1915* (NF).

### ﻿2. Sorbus arguta

Taxon classificationPlantaeRosalesRosaceae

T.T. Yu, Acta Phytotax. Sin. 8(3): 223. 1963.

83052A75-7109-586D-8F76-D08C1E3FC673

≡ Micromelesarguta (T. T. Yu) Mezhenskyj, NULESU Coll. Fruit Ornament. Pl.: 33. 2018.
≡ Wilsonariaarguta (T. T. Yu) Rushforth, Phytologia 100(4): 241. 2018.
= Ariayuarguta H. Ohashi et Iketani, J. Jap. Bot. 68(6): 361. 1993.

#### Type.

China. Sichuan: Pingshan, Chingping Shan, 1120 m, 26 May 1934, *T.H. Tu 5449* (holotype: PE[PE00934274]; isotype: PE[PE00934275])

#### Notes.

When describing *Sorbusarguta*, Yü designated “*T. H. Tu 5449*” at PE (Fig. 2B) as the holotype (Yü and Kuan 1963). It was accepted by Yü and Lu (1974), Gabrielian (1978), Phipps et al. (1990) and Lu and Spongberg (2003). Ohashi and Iketani (1993) transferred it to genus *Aria* and proposed a new name *A.yuarguta* H. Ohashi et Iketan (Ohashi and Iketan 1993: 361) for *A.arguta* had been already used by Roemer in 1847 for a different species. Mezhenskyj transferred it to *Micromeles* (Mezhenska et al. 2018: 33) and Rushforth (2018: 241) transferred it to *Wilsonaria*.

**Figure 3.**
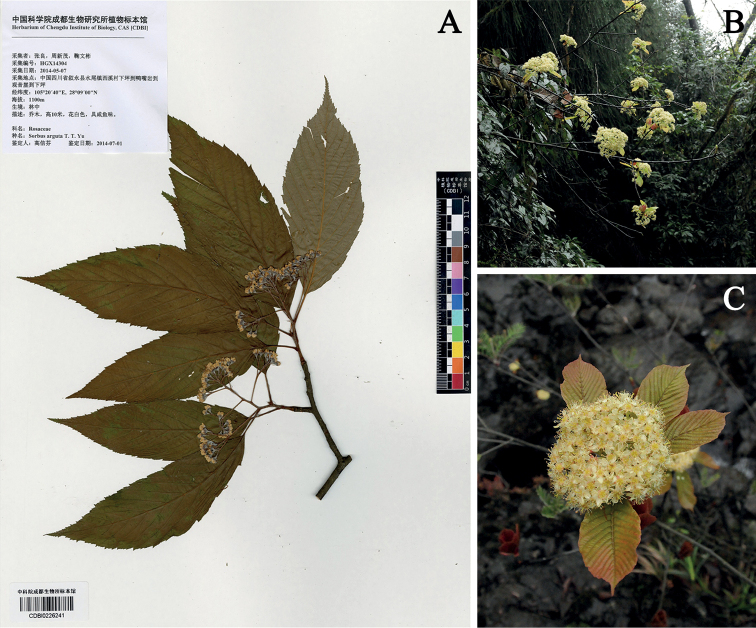
Corymbs of *Sorbusarguta* and *S.megalocarpa***A** flowering specimen of *Sorbusarguta* (CBDI0226241) collected by Liang Zhang, Xinmao Zhou and Wenbin Ju, 7 May 2014 **B***Sorbusmegalocarpa* at blossoming stage, 21 March 2021 **C** detail of the inflorescence of *S.megalocarpa* (**B**, **C** were taken by Tailun Hu).

However, Aldasoro et al. (2004) argued that: “*S.arguta is a minor variant of S.megalocarpa and does not deserve taxonomic recognition*”, and reduced it to a synonym of the later. A detailed study of the original material showed that *Sorbusarguta* is obviously different from *S.megalocarpa*. *Sorbusarguta* has oblong-ovate or ovate-lanceolate leaves with double serrate margins, relatively small corymbs (2–4 cm in diameter) with few flowers, and small sub-globose fruits (1–1.2 cm in diameter) with sparse lenticels, while *S.megalocarpa* has leaves with crenate-serrate margins, large corymbs (10–15 cm in diameter), many flowered (124–258 flowers per inflorescence), and much larger fruits with dense lenticels. Furthermore, *S.arguta* flowers after leaves are unfolded in early May (Fig. 3A, CBDI0226241), whereas *S.megalocarpa* flowers simultaneously with or before the leaves are unfolded in March (Fig. 3B, C). Therefore, *S.arguta* is treated as a distinct species here following Yü and Lu (1974), Gabrielian (1978), Phipps et al. (1990) and Lu and Spongberg (2003).

Representative specimens examined. China. Sichaun: Xinwen county, Xianfeng town, Monkey Bay, 1290 m, 12 May 1959, *Yibin wild economic plants collection team 0368* (CDBI); Xuyong county, Heishuihe Nature Reserve, 1500 m, 6 June 2007, *D.H. Zhu*, *Z.B. Feng*, *C. Zhang*, *F. Wang 20070776* (WCSBG); Xuyong county, Shuiwei town, Xixi village, 28°09'00"N, 105°20'40"E, 1100 m, 7 May 2014, *L. Zhang*, *X.M. Zhou*, *W.B. Ju HGX14304* (CDBI); Xuyong county, Shuiwei town, Xixi village, 28°08'01"N, 105°22'20"E, 1230 m, 29 July 2014, *W.B. Ju HGX14833* (CDBI).

## Supplementary Material

XML Treatment for Sorbus megalocarpa

XML Treatment for Sorbus arguta
